# The Predictive Value of Lung Ultrasound Score on Hemodynamically Significant Patent Ductus Arteriosus among Neonates ≤25 Weeks

**DOI:** 10.3390/diagnostics13132263

**Published:** 2023-07-04

**Authors:** Haifeng Zong, Zhifeng Huang, Bingchun Lin, Jie Zhao, Yongping Fu, Yanliang Yu, Hongyan Sun, Chuanzhong Yang

**Affiliations:** Department of Neonatology and Neonatal Intensive Care Unit, Affiliated Shenzhen Maternity and Child Healthcare Hospital, Southern Medical University, Shenzhen 518028, China

**Keywords:** lung ultrasound, score, patent ductus arteriosus, preterm infant, neonate

## Abstract

Lung ultrasound (LU) is increasingly used to diagnose and monitor neonatal pulmonary disorders; however, its role in hemodynamically significant patent ductus arteriosus (hsPDA) has not been elucidated. This prospective study investigated the predictive value of the LU score (LUS) for hsPDA in preterm infants with gestational age (GA) ≤ 25 weeks. Preterm infants with GA ≤ 25 weeks were enrolled in this study. LU was conducted on the fourth day of life (DOL). Six lung regions in every lung were scanned, with each region rated as 0–4 points. The performance of the LUS in predicting hsPDA among infants aged ≤25 weeks was analyzed by plotting the receiver operating characteristic (ROC) curve. A total of 81 infants were included in this study. GA, birth weight (BW), gender, Apgar score, delivery mode, antenatal steroids, meconium-stained amniotic fluid, premature rapture of membrane, and early-onset sepsis were not significantly different, but infants in the hsPDA group had increased LUS (38.2 ± 2.8 vs. 30.3 ± 4.3, *p* < 0.001) compared with non-hsPDA group. The area under the ROC curve (AUC) value of the LUS on the fourth DOL was 0.94 (95% CI: 0.93–0.99) in predicting hsPDA. The LUS threshold at 33 achieved 89% sensitivity and 83% specificity, with the positive and negative predictive values (PPV and NPV) being 87 and 86%, respectively. The LUS can predict hsPDA in extremely preterm infants at an early stage.

## 1. Introduction

For fetuses, lungs that are filled with fluid show great vascular resistance, while their systemic circulation exhibits a low vascular resistance; as a result, the fetal circulation displays a right-to-left shunting feature. After birth, adaptation of the circulatory system is decided by closure of the ductus arteriosus (DA) in the early days of life (DOL). As for preterm infants, their DA may usually not close due to smooth muscle immaturity or biochemical oxygen-sensing mechanisms [1]. The incidence of patent ductus arteriosus (PDA) is approximately 20–50% among newborns born with a gestational age (GA) of less than 32 weeks, while it is as high as 60% among those born with GA < 29 weeks [2,3]. Typically, decreased GA and birth weight (BW) indicate an increased PDA incidence and need for surgical treatment of PDA [4,5].

The maturation level is associated with the course of PDA closure. Hypoxia and immaturity of the ductal closure mechanism interfere with closure progress [6]. Persistent PDA may show clinical consequences determined by the left-to-right shunting level. Reduced pulmonary vascular resistance—in particular, for neonates with extremely low GA—will elevate the left-to-right shunting incidence via the ductus while increasing pulmonary blood flow, causing volume load into the left heart and pulmonary edema. The possible outcomes of left-to-right shunting induced by hemodynamically significant patent ductus arteriosus (hsPDA) are a higher risk of bronchopulmonary dysplasia (BPD), prolonged ventilation, necrotizing enterocolitis (NEC), acute renal failure, intraventricular hemorrhage (IVH), neurodevelopmental impairment, focal intestinal perforation, and even mortality [7,8].

At present, PDA is mainly assessed using echocardiography, which requires considerable expertise, and these skills cannot be acquired quickly. Lung ultrasound (LU) is used to diagnose and manage newborn diseases, and a guideline for LU has also been put forward for standardizing the approach [6]. LU can be performed at the bedside in an easy and rapid manner, which facilitates the rapid diagnosis of the disease and offers real-time data for pulmonary pathological features. In addition, LU exhibits an abrupt learning curve that can be easily mastered. In addition, agreement between observers is high across clinicians trained for LU [9,10,11]. In recent years, LU has been used as a “functional” tool to quantify pulmonary pathological features and predict clinical treatment at an early stage [11,12,13,14,15]. However, few studies have evaluated the hemodynamic status of the pulmonary circulation in extremely preterm infants with hsPDA using the LU score (LUS). Therefore, the present study assessed whether the LUS could be applied to predict hsPDA in preterm infants with GA ≤ 25 weeks.

## 2. Materials and Methods

### 2.1. Study Design

This study was approved by the Ethics Committee of the hospital (No. SFYLS2019119). Immediate families or guardians of children provided informed consent before LU. All methods were performed in accordance with the Declaration of Helsinki.

This prospective observational study (July 2019–June 2022) was conducted at Shenzhen Maternity and Child Healthcare Hospital affiliated with Southern Medical University, a hospital with an annual number of deliveries of nearly 20,000. LU was conducted in preterm newborns born at ≤25^+6^ weeks of GA who were admitted to the Department of Neonatology Intensive Care Units (NICU) after obtaining consent from their parents before and after delivery. The inclusion criteria were as follows: premature infants with a GA ≤ 25^+6^ weeks and those born in our hospital. Newborns with congenital heart disease (CHD) evidenced by cardiac ultrasound (with the exception of patent foramen ovale and PDA), those developing congenital abnormalities or additional chromosome abnormalities detected before delivery, and those showing pulmonary hemorrhage or pneumothorax were excluded from the present work. In addition, the potential causes of extravascular lung fluid increase were also considered, for example, hypoalbuminemia, premature rupture of membrane (PROM) > 18 h, early-onset sepsis (EOS), meconium-stained amniotic fluid (MSAF), mechanical ventilation (MV), acute kidney injury, and volume overload.

Infants in the NICU underwent echocardiographic examinations on the fourth DOL by a sonographer, and echocardiographic examinations followed the LU scanning. The following examinations were performed: narrowest ductal diameter determined based on parasternal short axis view; ductal flow velocity determined according to ductal flow based on parasternal short axis view; ratio of left atrium-to-aortic root determined based on parasternal long axis; and descending aortic flow evaluated distal to DA origin (ductal ampulla). In this study, non-identifiable flow within the DA detected based on color Doppler was deemed as ductal closure [16].

In our NICU, the preterm infant has an umbilical artery or peripheral artery catheter for invasive monitoring of blood pressure (BP) early postnatally, with the tip lying in the descending aorta near the DA insertion. Invasive BP was monitored following standard neonatal intensive care using a Dräger Vista 120 patient monitoring system (Dräger Medical GmbH, Luebeck, Germany). In addition, this study localized the BP transducer to the cardiac level. To guarantee a favorable quality signal, a visual assessment of the BP waveforms was performed on the monitor screen.

### 2.2. Hemodynamically Significant PDA

Integrative echocardiographic examination includes the evaluation of ductal features, pulmonary over-circulation parameters, and systemic hypoperfusion signs. The hsPDA displays clinical characteristics of elevated oxygen demand and respiratory support, frequency and degree of apnea, tachypnea, tachycardia, feeding intolerance, abdominal distension, radiological findings showing pulmonary edema and cardiomegaly, oliguria, and diastolic/mean hypotension with or without metabolic acidosis that requires cardiotonic or vasopressor agents. A thorough echocardiographic assessment was conducted to eliminate pulmonary hypertension or possible congenital heart defects before any intervention to close the PDA [16,17]. This work deemed hsPDA as the open DA showing a predominant left-to-right flow and either: (1) ductal diameter ≥ 1.5 mm; (2) left-atrium-size-to-aortic-root-diameter ratio (LA/Ao) ≥ 1.5; and (3) reversed or no flow in the descending aorta in the diastolic period observed from a suitable view [18,19]. 

### 2.3. LU and LUS

LU was scanned on the fourth DOL by an experienced sonographer, using a standardized protocol that was blinded to the clinicians. Two evaluators, who were blinded to the clinical details, were responsible for assessing each image. The hsPDA was determined by a neonatologist who was blinded to the LUS. Moreover, LU was performed using a commercial portable US device (M9, Mindray, Shenzhen, China) with the “hockey stick” microlinear probe (L16-4Hs, 3.5–16 MHz). 

Lung partition: This study scanned the anterior, lateral, and posterior regions of the chest wall. The hemithorax was divided into six areas (single focus at the pleural line, B-mode), including the upper and lower anterior/lateral/posterior areas [20]. The scanning process was performed down the midclavicular, paravertebral, and midaxillary lines. Horizontal and longitudinal scans were performed and harmonics were not chosen. Later, one sonologist experienced in the LUS of the children was responsible for examining each scan. 

**Figure 1:**
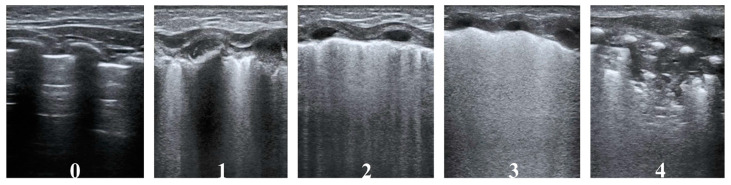
Description of the LUS. The 12-zone scanning protocol was adopted. For each lung area, a score from 0 to 4 was assigned. Score values corresponded to 5 different patterns, as shown in the ultrasonograms. The score was given as follows: 0 points (presence of <3 well-spaced B-lines); 1 point (presence of ≥3 well-spaced B-lines); 2 points (B-lines are difficult to count, or partially coalescent); 3 points (fully coalescent B-Lines, with or without minor consolidations limited to the subpleural space); 4 points (extended consolidations).

A 5-grade scale (Table 1) was used to grade every area, which was modified based on Brat et al. [11]. Two reviewers were responsible for describing the type and size upon the completion of scanning. Each area was assigned an LUS of 0–4 points: 0 indicates the presence of <3 well-spaced B-lines, indicating a relatively well-aerated lung; 1 indicates that there are ≥3 well-spaced B-lines; 2 indicates that B-lines are difficult to count, or partially coalescent; 3 indicates the presence of fully coalescent B-Lines, with/without minor consolidations restricted to the subpleural space; and 4 indicates the presence of extended consolidations. Representative images are shown in Figure 1. The scores of the 12 areas were added to obtain the total LUS (range, 0–48). An additional grade of “B-lines are difficult to count, or partially coalescent” (score “2”) was added in this work, so as to obtain more precisely and continuously assess pulmonary lesion severity. Disagreements were resolved by consensus.

### 2.4. Sample Size and Statistical Analysis

SPSS23.0 (IBM Corporation, New York, NY, USA) was used for statistical analysis. Demographic information, clinical features, and echographic features were summarized. First, the Kolmogorov–Smirnov test was applied to test data normality, which was then represented as the mean (standard deviation, SD) or median (interquartile range, IQR). Univariate analyses, including the Student’s *t*-test, chi-square test, Mann–Whitney test, and Fisher’s exact test, were conducted between the hsPDA and non-hsPDA groups. The predictive performance of the LUS in hsPDA was analyzed by plotting the receiver operating characteristic (ROC) curve, and areas under the ROC curves (AUCs) and cutoff values were determined. Each test was two-sided, with *p* < 0.05 indicating statistical significance.

This study determined the sample size as follows. The incidence of hsPDA 1 year prior to this study was approximately 55% in the NICU, as indicated by our predetermined inclusion criteria. The sample size was calculated according to the simple random sampling formula, with α = 0.05 and δ = 0.12. A design effect of 1.2 yielded an effective sample size of 80, indicating that 80 newborns were required. Analyses were performed using power analysis and sample size (PASS) software (version 15.0, NCSS, LLC, Kaysville, UT, USA, ncss.com/software/pass).

## 3. Results

During the study period, 92 eligible preterm infants with a GA ≤ 25^+6^ weeks were admitted to our center. However, 3 patients died before the fourth DOL, while 8 were eliminated in line with the exclusion criteria (*n* = 5 due to clinical instability on the fourth DOL and *n* = 3 due to inadequate images). Ultimately, 81 infants were included in this study. Table 2 displays the demographic data of the enrolled patients and the measurements summary.

There were 35 cases in the non-hsPDA group, with a median GA of 25 weeks (IQR, 24.4–25.4), mean BW of 718 ± 109 g, and mean LUS of 30.3 ± 4.3. There were 46 cases in the hsPDA group, with a median GA of 24.8 weeks (IQR, 24.2, 25.2), mean BW of 688 ± 110 g, and mean LUS of 38.2 ± 2.8. There were no cases of hydrops fetalis in this study. There were no significant intergroup differences in GA, BW, sex, Apgar score, delivery mode, antenatal steroids, premature rupture of membrane (PROM) > 18 h, EOS, or MSAF between the two groups, whereas the LUS was significantly different between the two groups. Infants in the hsPDA group had an increased LUS (38.2 ± 2.8 vs. 30.3 ± 4.3, *p* < 0.001) compared with the non-hsPDA group. The average time taken for LU scanning was four minutes, compared to six minutes for cardiac ultrasound examination. No prophylactic indomethacin or ibuprofen was administered. All infants in the hsPDA group received medical treatment, and 14 of them received surgical treatment.

**Figure 2:**
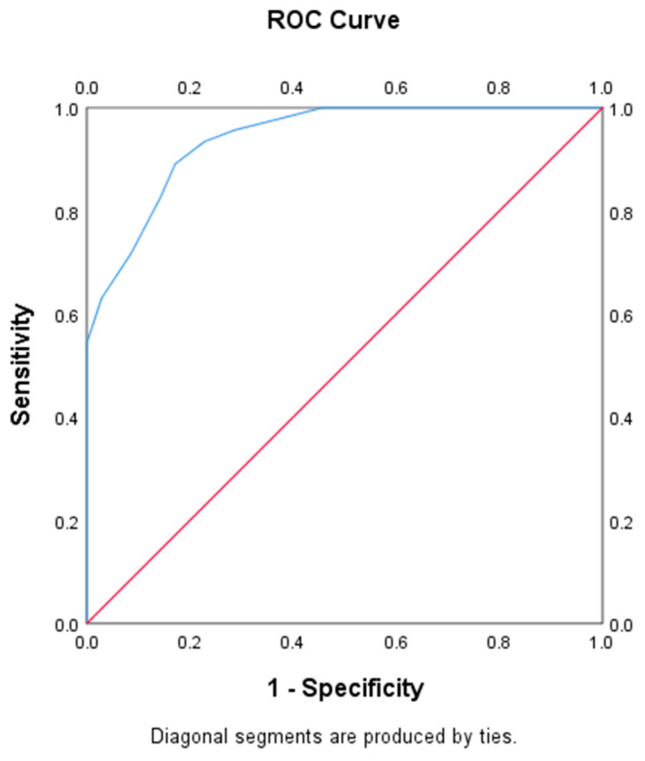
The constructed ROC curve for the prediction of hsPDA by LUS. LUS, lung ultrasound score; hsPDA, hemodynamically significant patent ductus arteriosus; ROC, receiver operating characteristic.

ROC curve analysis was also performed to assess whether the LUS could predict hsPDA. The result revealed that the AUC was 0.94 (95% confidence interval [CI]: 0.89–0.99; *p* < 0.001), while when the LUS threshold was set at 33, it achieved a sensitivity of 89%, specificity of 83%, and positive/negative predictive value (PPV/NPV) of 87% and 86%, respectively (Figure 2). Finally, the interobserver consistency (Cohen’s k = 0.82, ICC = 0.977) between the two assessors for the LUS assessment was good.

## 4. Discussion

PDA is the most frequently observed cardiac disease among newborns with GA ≤ 25 weeks. Accurate early prediction of hsPDA can assist clinicians in identifying high-risk newborns, possibly benefiting from targeted treatment. Echocardiography is the conventional evaluation method and requires considerable expertise and experience. In this prospective study, it was found that the LUS on the fourth DOL provided an excellent prediction of hsPDA in preterm newborns with GA ≤ 25 weeks. The LUS is clearly different in hsPDA than in non-hsPDA. To the best of our knowledge, the present study is the first to prospectively suggest that the LUS can help predict hsPDA in preterm infants with GA ≤ 25 weeks with a very high degree of precision.

Lower GA and BW indicate higher incidence rates of PDA and hsPDA [5,21]. For preterm newborns with GA ≤ 28 weeks, the PDA (i.e., open DA 3 days after delivery) incidence was >50% [22]. Spontaneous PDA closure occurs at a lower rate in preterm newborns with a GA < 28 weeks. The time needed for closure is inversely proportional to birth GA, and certain vessels may be closed in months to years. Historical practice induces surgical or medical treatment among 60–70% of preterm newborns with GA < 28 weeks [23]. Persistent hsPDA in preterm newborns may lead to certain clinical results. Reducing pulmonary vascular resistance, particularly for neonates with extremely low GA, will lead to an elevated incidence of left-to-right shunting via the ductus while increasing pulmonary blood flow, which can cause pulmonary edema, respiratory deterioration, and reduced blood flow in the kidney, brain, and gastrointestinal tract. Pulmonary edema is a principal manifestation of hemodynamic instability caused by hsPDA [24].

LU is a convenient bedside imaging technique with high safety, non-invasiveness, ease of operation, and no ionizing radiation. In the last 10 years, LU has been widely adopted for the diagnosis and monitoring of neonatal pulmonary lesions. Moreover, LU has been used as a “functional” tool to quantify pulmonary pathological features and predict clinical treatment [11,12,13,14,15,25]. Scores have been proposed to determine the implementation of certain therapies. In recent years, semi-quantitative LUS has been utilized to predict surfactant treatment demand, which is necessary for respiratory support, and BPD progression [26,27]. However, to date, there have been no reports of the use of the LUS to guide PDA management.

Brat et al. first described the classic neonatal LUS strategy [11]. Each lung was classified into three areas, namely, upper anterior, lateral, and lower anterior, with 0–3 points in every area, where 0 stands for A-lines alone; 1 indicates that there are ≥3 well-spaced B-lines; 2 suggests that there were coalescent and crowded B-lines; and 3 indicates the extension of consolidations. The posterior lung zones were not evaluated in this scoring system. Similarly, to additional inflammatory pulmonary lesions such as acute respiratory distress syndrome (RDS) and bronchiolitis, the severely affected areas are the dependent lung zones, and the posterior lung fields are generally less aerated in extremely preterm infants [25]. At present, some distinct partitioning strategies have been proposed for the LUS, with 6- and 12-zone approaches extensively utilized [28]. This study utilized a 12-zone approach to cover the entire lung field surface. It was assumed that assessing a lung aeration-dependent distribution was added to LU prediction accuracy; therefore, this study introduced posterior lung field scanning. In addition, most previous studies adopted a 4-grade rating scale scoring system that neglected the common situations in clinical practice in which B-lines are difficult to count or partially or fully coalescent. In our modified scoring system, common situations are given specific instructions. Eventually, the current modified LU scoring strategy includes posterior lung fields as well as the 5-grade rating scale, but not the 4-grade one.

A previous study showed that the number of B-lines can reflect the pulmonary water content [12]. The present study adopted the LUS to assess extravascular pulmonary water content in newborns with hsPDA. Zhao et al. studied 94 cases of low-birth-weight infants and assessed PDA using the LUS graded by Brat et al. [29]. The results showed that the AUC was 0.668, while the sensitivity and specificity for predicting PDA were 50% and 76.4%, respectively. In addition, the ROC curve showed low sensitivity, which did not accurately reflect PDA. In our study, the LUS of the hsPDA group dramatically increased relative to that of the non-hsPDA group, with an AUC of 0.94, while sensitivity and specificity were 89% and 83%, respectively, and the LUS might be used to predict hsPDA. There might have been two main reasons for this. One reason is the subjects. The subjects in their study were just premature infants with a birth weight less than 2500 g, while the subjects in our study were preterm infants with GA ≤ 25 weeks. The other reason is the LUS strategy. In the previous study, each lung was divided into 3 areas and the 4-grade rating scale was used, whereas in the present study, each lung was divided into 6 areas and the 5-grade rating scale was adopted.

The LU scan was conducted on the fourth DOL. Owing to the rapidly declining pulmonary artery pressure following birth, it remained at a normal level at 72 h [30]. In fact, infants with GA ≤ 25 weeks need a longer time for successful transition from intrauterine to extrauterine life. For fetuses, lungs that are filled with fluid show great vascular resistance, while their systemic circulation exhibits a low vascular resistance; as a result, the fetal circulation displays a right-to-left shunting feature. After birth, systemic blood pressure rises in newborns; meanwhile, lung expansion decreases pulmonary artery pressure and pulmonary vascular resistance. In these infants, lung aeration and liquid clearance are delayed because of lung immaturity, weak respiratory effort, immature epithelial sodium channels, and decreased concentrations of catecholamines and other hormones in the first three days after birth [31,32]. In addition, pharmacological therapy can be initiated when hsPDA is detected within seven DOL [8]. A recent study showed that extremely preterm infants receiving treatment within six DOL are associated with markedly decreased BPD and/or death [33]. We selected extremely preterm newborns with GA ≤ 25 weeks. This is because few infants require invasive mechanical ventilation with GA ≥ 26 weeks, and there is a relatively low incidence of BPD and related complications.

In this study, each hemithorax was classified into six regions. A 5-grade scale (Table 1) was used to grade every lung region. LUS 4 means extended consolidations in a lung region. According to our experience, extended consolidations are often seen in severe RDS in extremely preterm infants early after birth [9]. Such findings are more common in bilateral posterior lungs. However, the incidence of RDS is high in these infants, the majority of whom have been administrated exogenous pulmonary surfactant after birth. In addition, lung ultrasound scanning was conducted on the fourth DOL when the influence of RDS is relatively small. These things considered, infection, MV, meconium aspiration syndrome (MAS), gastroesophageal reflux, and pulmonary hemorrhage can also be manifested as extended consolidations. In this study, EOS, PROM, MSAF, and MV were all considered and did not differ significantly between the non-hsPDA group and the hsPDA group.

Will extended consolidations in a lung area delay ductus closure? There are still difficulties in reaching a conclusion. Firstly, the GA of infants was ≤25 weeks in this study, and in our experience, extensive consolidation is very common in these infants. In addition, extended consolidation is often a consequence of hsPDA rather than a cause of hsPDA. Finally, extended consolidation can cause hypoxemia, which may delay ductus closure. More research is needed in the future to investigate the impact of extended consolidation on ductus closure in clinical practice.

Echocardiography is the conventional evaluation method for PDA. To properly utilize echocardiography results in clinical decisions, it is necessary to possess specific echocardiography expertise and substantially understand cardiovascular disease. Therefore, physicians involved in echocardiography research must possess extensive skills that cannot be acquired rapidly. Specifically, the physician in charge of echocardiography implementation and interpretation should focus on relevant techniques for evaluating data quality and making appropriate conclusions in diagnosis [34]. The shunt volume across the DA is usually determined by pulmonary and systemic vascular resistance, adaptation of the myocardium to elevated shunt volume, and DA size [16]. Infant activity can also affect the ductal blood flow velocity of shunting [35]. In contrast, LU can be conducted at the bedside in a rapid and easy manner, which shows an abrupt learning curve and can be easily learned [36]. Moreover, interobserver consistency is high across clinicians trained for LU [10,11]. 

According to our study, the LUS allows for semi-quantitative evaluation of pulmonary edema levels, which can predict hsPDA at an early stage of life in extremely preterm infants. Some limitations of the present study should be noted. First, no consensus has been reached regarding the LUS strategy. There are variations in the LUS system for preterm infants among clinical units. Second, the present unicentric study had a low sample size. Multicenter studies with larger sample sizes are needed to validate our findings. Third, the LUS is a semi-quantitative evaluation of lung function, and the score may be impacted by the operator’s subjective factors.

## 5. Conclusions

In conclusion, the LUS can predict hsPDA in extremely preterm neonates at an early stage. 

## Figures and Tables

**Table 1:** Lung ultrasound score, with a score of 0–4 is recorded at each scan region. Scores were defined according to the following criteria. The sum of the score yields a total score denoting the severity of the lung.

Score	Sonographic Appearance
0	presence of <3 well-spaced B-lines
1	presence of ≥3 well-spaced B-lines
2	B-lines are difficult to count, or partially coalescent
3	fully coalescent B-Lines, with or without minor consolidations limited to the subpleural space
4	extended consolidations

**Table 2:** Baseline characteristics of the study population.

	Non-hsPDA Group (*n* = 35)	hsPDA Group (*n* = 46)	*p* Value
GA, weeks	25.0 (24.4, 25.4)	24.8 (24.2, 25.2)	0.058
Birth weight, g	718 ± 109	688 ± 110	0.069
Male	24 (68.6)	24 (52.2)	0.173
Apgar score 1 min	6 (5, 8)	6 (5, 8)	0.427
Apgar score 5 min	9 (8, 10)	9 (8, 10)	0.972
Spontaneous vaginal delivery	26 (74.3)	36 (78.3)	0.676
Antenatal steroids, full course	23 (65.7)	27 (58.7)	0.520
MSAF	1 (2.9)	1 (2.2)	1.000
PROM > 18 h	8 (22.9)	14 (30.4)	0.448
EOS	10 (28.6)	12 (26.1)	0.803
EPS	32 (91.4)	43 (93.5)	0.998
MV	29 (82.8)	38 (82.6)	0.977
LUS	30.3 ± 4.3	38.2 ± 2.8	0.000

Values are expressed as mean ± standard deviation, median (25th; 75th centile) or number (%). EOS, early-onset sepsis; EPS, exogenous pulmonary surfactant; GA, gestational age; LUS, lung ultrasound score; MV, receiving mechanical ventilation on the fourth day of life; PROM, premature rupture of membrane; MSAF, meconium-stained amniotic fluid.

## Data Availability

All data utilized in the present study can be obtained from the corresponding authors upon request.
